# Y-Chromosomal insights into the paternal genealogy of the Kerey tribe have called into question their descent from the Stepfather of Genghis Khan

**DOI:** 10.1371/journal.pone.0309080

**Published:** 2024-09-04

**Authors:** Maxat Zhabagin, Alizhan Bukayev, Zhanargul Dyussenova, Altyn Zhuraliyeva, Assel Tashkarayeva, Aigul Zhunussova, Baglan Aidarov, Akynkali Darmenov, Ainur Akilzhanova, Uli Schamiloglu, Zhaxylyk Sabitov

**Affiliations:** 1 National Center for Biotechnology, Astana, Kazakhstan; 2 Nazarbayev University, Astana, Kazakhstan; 3 International Science Complex “Astana”, Astana, Kazakhstan; 4 Research Institute for Jochi Ulus Studies, Astana, Kazakhstan; 5 Astana International University, Astana, Kazakhstan; 6 Karaganda Academy of the Ministry of Internal Affairs of the Republic of Kazakhstan named after Barimbek Beisenov, Karaganda, Kazakhstan; 7 National Laboratory Astana, Astana, Kazakhstan; 8 L.N. Gumilyov Eurasian National University, Astana, Kazakhstan; 9 Kazak Historical Society, Astana, Kazakhstan; Xiamen University, CHINA

## Abstract

The Kerey is one of the prominent Kazakh tribes and has long been a subject of ethnographic scrutiny, with a lack of consensus on its origin and traditional genealogy. Their historical significance, intertwined with the emergence of the empire established by Genghis Khan, necessitates a comprehensive understanding of their genetic history. This study focuses on unraveling the genetic heritage of the Kerey tribe. We conducted a comprehensive analysis of Y-chromosome data from genetic genealogy as citizen science and genetic screening of 23 Y-STRs and 37 Y-SNPs on 207 males from the Kerey tribe within academic science. Our results reveal two prevalent phylogenetic lineages within the C2a1a3a-F3796 haplogroup, also known as the C2*-Star Cluster (C2*-ST), which is one of the founding paternal lineages of the ancient Niru’un clan of the Mongols: C2-FT411734 and C2-FT224144, corresponding to the Abak and Ashamaily clans. While indicating a common male ancestry for them, our findings challenge the notion that they are full siblings. Additionally, genetic diversity analysis of the Y-chromosomes in the Kerey tribe and Kazakhs confirms their kinship with the Uissun tribe but refutes the claim of the Abak clan’s progenitor originating from this tribe. Furthermore, genetic evidence fails to support popular historical and ethnographic hypotheses regarding the Kerey tribe’s kinship with the Uak, Sirgeli, Adai, Törtkara, Karakerey, and Kereyit Kazakh tribes. The absence of a genetic paternal connection with the Kereyt tribe raises doubts about the genealogical link between the Kerey tribe and the stepfather of Genghis Khan.

## Introduction

The examination of the Y-chromosome has emerged as a prominent field within human population genetics [1]. In addition, it has gained widespread popularity as a genetic genealogy tool in modern times [2]. The utility of this tool is particularly evident in interdisciplinary research focused on verifying historical and ethnographic data pertaining to the ancestral lineage of clans and tribes [3,4]. Also, it proves valuable in the process of reconstructing the patrilineal genealogy of these familial lineages [5].

The Kazakhs are a Turkic-speaking indigenous group of Central Asia comprised of many patrilineal tribes of varied origins [6]. Some tribes’ memories are still maintained in the traditional genealogy of the Kazakh "Shezhire". It has an essential part in Kazakh ethnic identification [7]. The Kazakhs are made up of approximately 20 big tribes and 200 clans nowadays. The Shezhire genealogical structure and clan distribution are valuable historical and ethnographic data, as well as an intriguing item for investigating the genetic structure of the Kazakh population [8,9].

The Kerey is considered to be one of the prominent Kazakh tribes, with its historical roots often linked to the medieval Kereyt (Keraites) tribe. This particular tribe established their settlement in the region near the Great Wall of China and the Mongolian plain, specifically in the upper areas of the Onon, Kerulen, and Orkhon rivers [6]. One of the notable figures in their history is Wang Khan, who is also well known as Toghrul. He is renowned for his close relationship with Yesugei Baghatur, the chief of the Kiyat-Borjigin clan, whom he considered a blood brother. Following Yesugei’s demise, Toghrul assumed the role of Genghis (Chinggis) Khan’s stepfather, becoming both his ally and later his adversary [10]. Nevertheless, it is worth noting that Chinese texts employ distinct logographic characters when referring to the Kerey and Kereyit tribes, hence raising questions regarding their shared ancestry [11].

The modern census of the population of Kazakhstan does not account for the number and location of tribes. According to the most recent historical data from the census of the late 19th century, the Kerey tribe primarily settled in the areas of Petropavlovsk (over 20,000 people), Omsk (more than 13,000 people), Karkaralinsky (roughly 12,000 people), Kostanai (exceeding 8,000 people), Semey (roughly 6,000 people), and Zaisan (about 5,000 people). The total Kerey population was between sixty-five and seventy thousand [11]. Thus, the principal Kerey settlements are in northern and eastern Kazakhstan.

**Fig 1:**
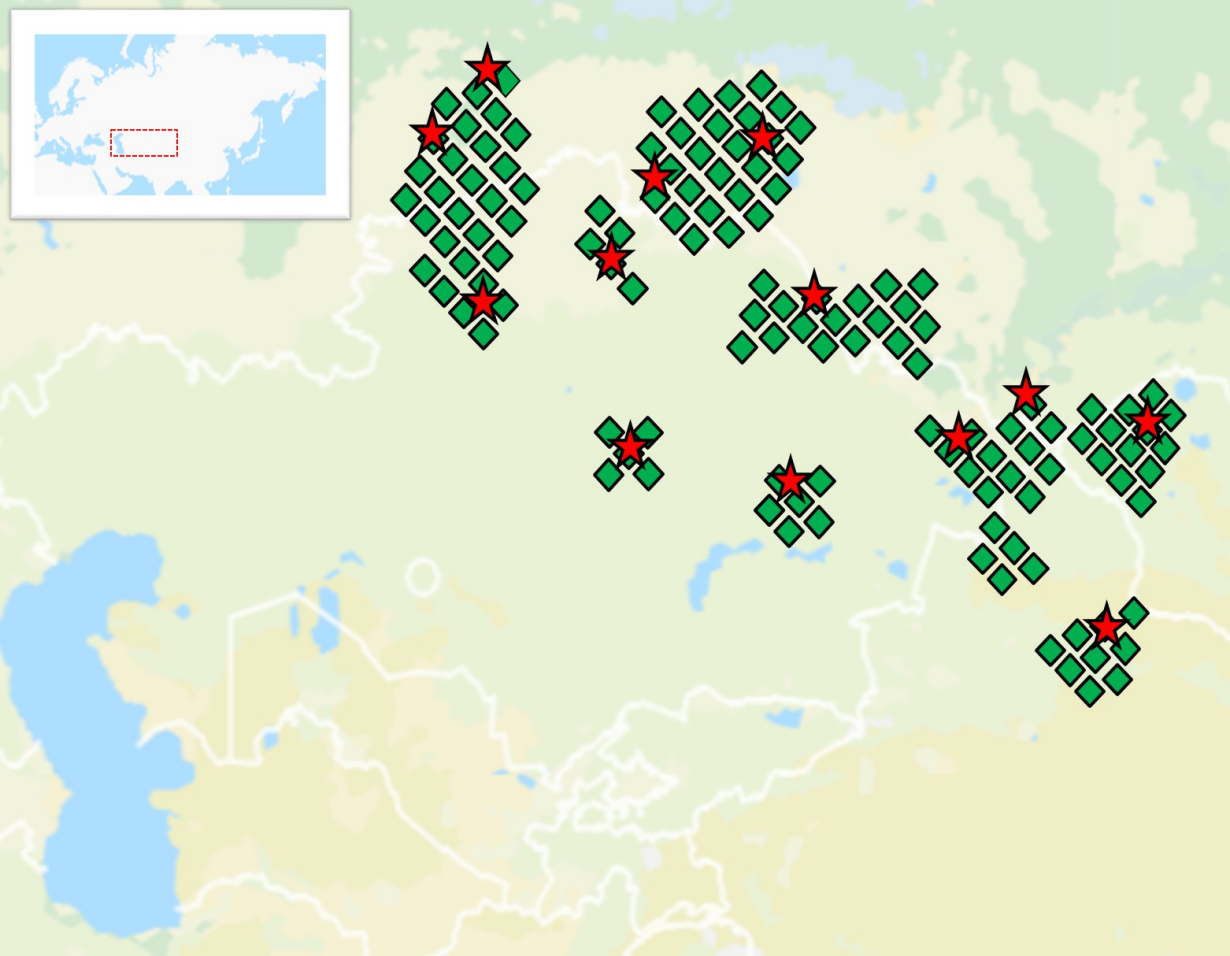
The settlement of the Kerey tribe during the early 20th century. The green rhombuses serve as indicators of the settlement regions for the tribe. The red stars show the geographical locations from where the ancestors of the persons studied in this study originated, specifically tracing back three generations. The base map was sourced and adopted from OpenStreetMap and OpenStreetMap Foundation, which is made available under the Open Database License.

According to the most recent census, there are 16 million Kazakhs, of which 13.5 million reside in Kazakhstan. The combined rural areas of the northern and eastern regions account for around 17% of the total population in Kazakhstan (https://stat.gov.kz). Recent estimates indicate that the Kerey tribe in Kazakhstan consists of more than 350,000 people [12]. Furthermore, it is noteworthy that the Kerey population is predominantly concentrated among the Kazakhs residing in Western Mongolia’s Bayan-Ulgii aimag (province), the north-western region of China encompassing the Xinjiang Uygur Autonomous Region and Gansu Province, and the southern area of Russia’s West Siberian Plain (Fig 1). The current population of Kazakhs residing in Western Mongolia is estimated to be 121,000 people [13]. Additionally, it has been reported that there are somewhere around 1.6 million Kazakhs residing in northwest China [14]. Lastly, it has been indicated that the number of Kazakhs living in Russia is around 600,000 (https://rosstat.gov.ru/vpn/2020). Fig 1 schematically represents the settlement of the Kerey tribe based on census data from the early 20th century [15], complemented by information from the Historical Atlas of Central Asia [16].

Based on Kazakh genealogical records, it is observed that the Kerey tribe can be categorized into two major clans, namely the Abak and Ashamaily. On the basis of a particular interpretation of the Shezhire, it is posited that the Abak and Ashamaily share a common male ancestor and are considered siblings [17]. Contrary to alternative accounts, it has been argued that the Abak were not biologically related to the Ashamaily [11,18]. Various ethnographic accounts indicate that the overall structure and genealogical connections of affiliations vary among different versions.

Drawing on the field data obtained during ethnographic expeditions undertaken from 1956 to 1973, it is evident that the Ashamaily clan encompasses four separate lineages, specifically Syban, Balta, Koshebe, and Taryshy. The Abak clan is composed of eleven distinct lineages [19], specifically Zhantekei, Zhadik, Karakas, Sherushi, Zhastaban, Itely, Molky, Konsadak, Merkit, Itemgen, and Sarbas. Some authors have posited that the Abak clan encompasses other lineages such as Shimoyn, Shubaraigyr, and Kultaibolat [20–22]. Nevertheless, the precise correlation among all these lineages remains uncertain.

The Kazakh population exhibits a characteristic practice of patrilineal inheritance in relation to clan membership. The practice of "clan" inheritance has made it easier to use Y-chromosome polymorphism as a technique to verify genealogical lineage and examine its origins [23]. Through the incorporation of historical and ethnographic data, it becomes possible to reconstruct the genetic history of a certain population. Presently, research efforts focused on Y-chromosome polymorphism span a broad range of Y-chromosome varieties within the analytical framework. The presence of quickly evolving Y-STR loci has a significant role in facilitating the emergence of closely related males [24]. The examination of variability among clans holds significance in the fields of forensics and worldwide genetic genealogy assessments and findings. The use of next-generation sequencing (NGS) for the sequencing of Y-chromosomes has yielded significant discoveries in the form of several novel subbranches and Y-SNPs. Some studies demonstrated that these genetic variations are frequently specific to particular populations [25,26]. Citizen science, comprising a network of genealogists and amateur volunteers, significantly contributes to this cause through the research of Y chromosome polymorphisms using commercially available assays. For example, the FamilyTreeDNA platform has gathered around 50,000 subbranches and 460,000 variations of Y chromosomes by employing the BigY700 Y-chromosome deep sequencing product (https://www.familytreedna.com/public/y-dna-haplotree).

More than ten years ago, Y-chromosome polymorphism was used to study the Kerey tribe for 17 Y-STRs and 10 Y-SNPs [27]. The utilization of this approach for studying Kazakh tribes was unprecedented. Subsequent research has established that the Kerey tribe has a strong founder effect within the C2-M217 haplogroup. The prevalence rate of this phenomenon is reported to be 76.5% among the Kereys in Kazakhstan [27]. Similarly, among the Kazakhs residing in Aksai, located in Gansu Province, China, the frequency of occurrence is estimated to be 80% [28,29]. The specific subbranches of the C2-M217 haplogroup that are found within the tribal affiliations of the Kerey tribe have yet to be determined. There is a study that provided evidence of differentiation between the Abak and Ashamaily at the DYS448 loci, with respective values of 23 and 22 [27]. It is not known which lineages were included in these studies. Does the differential alone pertain to all the lineages within these two clans? Does differentiation occur at the level of Y-SNPs? Are there any genetic foundations for historical and anthropological theories concerning the interconnections between the Kerey tribe and other Kazakh tribes?

In the context of this research, we sought to address these inquiries by examining the polymorphism of the Y-chromosome within two distinct clans of the Kerey tribe: Ashamaily and Abak.

## Materials and methods

### Samples

A total of 207 Kazakh men who met the criteria for good health were chosen as participants in our study, and their venous blood samples were collected during several fieldwork sessions conducted in Kazakhstan from15 November 2020 to 15 November 2021. The criteria for selection included the individual’s affiliation with the Kerey tribe, their understanding of their clan, and the birthplace of their grandpa, as well as the removal of cousins within three generations in the paternal lineage from the sample. Every individual chosen for the study provided their written informed consent to partake in the research and completed an anthropological questionnaire. The blood sampling procedure and other research methodologies have undergone prior evaluation and received approval from the Local Ethical Commission at the National Center for Biotechnology (№5 by 16 October 2020). The study encompassed the progeny of individuals who migrated three generations ago to Kazakhstan (N = 158), Mongolia (N = 21), China (N = 19), and Russia (N = 9) (Fig 1). The present study encompasses the clan connections of the Ashamaily (N = 154) and Abak (N = 53), comprising four lineages of Ashamaily and ten lineages of Abak. Additional information regarding the ethical, cultural, and scientific considerations specific to inclusivity in global research is included in the (S1 Checklist).

### DNA analysis

The process of extracting genomic DNA from venous blood was conducted using the Wizard (R) Genomic DNA Purification Kit (Promega, USA). The concentration of DNA was measured using a Quantus Fluorometer (Promega, USA) and the QuantiFluor (R) ONE dsDNA System kit (Promega, USA). The determination of DNA quality was conducted using NanoDrop One equipment manufactured by ThermoFisher Scientific, United States. The amplification of 23 Y-STRs, specifically DYS576, DYS389I, DYS448, DYS389II, DYS19, DYS391, DYS481, DYS549, DYS533, DYS438, DYS437, DYS570, DYS635, DYS390, DYS439, DYS392, DYS643, DYS393, DYS458, DYS385a, DYS385b, DYS456, and YGATAH4, was conducted using the PowerPlexY23 amplification kit manufactured by Promega, USA. The amplification process was carried out on a SimpliAmp Thermal Cycler (ThermoFisher Scientific, USA). The examination of amplicons was conducted using an 8-capillary 3500 Genetic Analyzer manufactured by Thermo Fisher Scientific, United States. The phoregrams were subjected to analysis using the GeneMapper IDx v.1.4 software (ThermoFisher Scientific, USA), following the guidelines provided by the reference allelic ladder. The determination of haplogroups was accomplished through the utilization of haplotypes within the Nevgen Y-DNA haplogroup predictor tool, accessible at the following URL: https://www.nevgen.org/. The Y-chromosome haplogroups were analyzed by means of the web platform FamilyTreeDNA Discover (https://discover.familytreedna.com/), in conjunction with citizen science data. Subsequently, the genotyping of a set of 37 candidate Y-SNPs (M130, M217, F1756, Y148084, FGC28850, BY187593, M48, F5485, SK1066, Y15844, Y15552, F12970, F9766, F1918, F4002, F5481, BY182928, Y12782, Y20797, Y20086, FT224144, F18202, ZQ506, FT411734, FT250737, FGC29011, F1067, M407, ZQ402, F8465, M285, M253, M267, M231, M175, M198, M343) was performed on the QuantStudio5 instrument (ThermoFisher Scientific, USA) using TaqMan assays (ThermoFisher Scientific, USA).

### Statistical methods

The haplotype/allele frequencies, haplotype diversity (HD), haplotype match probability (HMP), discrimination capacity (DC), and forensic parameters were computed following the methodology described in our recent research [30]. The STRAF 1.0.5 software (http://cmpg.unibe.ch/shiny/STRAF/) [31] was utilized for these calculations. The Y-chromosome STR haplotypes were subjected to comparison using the Haplomatch tool [32]. To conduct a comparative analysis, we compiled a comprehensive dataset consisting of previously published information (N = 3856 haplotypes for 17 Y-STRs) derived from population samples of Kazakhs [30,33–36] and various Kazakh tribes [9,23,27,28,37–39]. Phylogenetic networks were constructed utilizing haplotypes derived from 15 Y-STRs in comparison with existing data, and 21 Y-STRs based on our own data, using the Network and Network Publisher software applications (http://www.fluxus-engineering.com). These networks were constructed employing Reduced-Median algorithms [40]. The numerical value representing the repetition of DYS389I was removed from the numerical value representing the repetition of DYS389II. The locus DYS385a/b was excluded.

## Results and discussion

### Paternal genetic portraits of Kerey tribe

A set of 23 Y-STRs and 37 Y-SNPs linked to Y-chromosome haplogroups were used to look at the genetic makeup of 207 people in the Kerey tribe. The portrait is also situated within the framework of the clannish affiliations of the Ashamaily (N = 154) and Abak (N = 53). The Ashamaily are characterized by the presence of four lineages, while the Abak are characterized by the presence of ten lineages. The portrait of the Kerey tribe encompasses individuals whose forebears, three generations prior, originated from Kazakhstan (N = 158), Mongolia (N = 21), China (N = 19), and Russia (N = 9), aligning with the geographical regions where the Kerey tribe is known to have settled (Fig 1).

**Fig 2:**
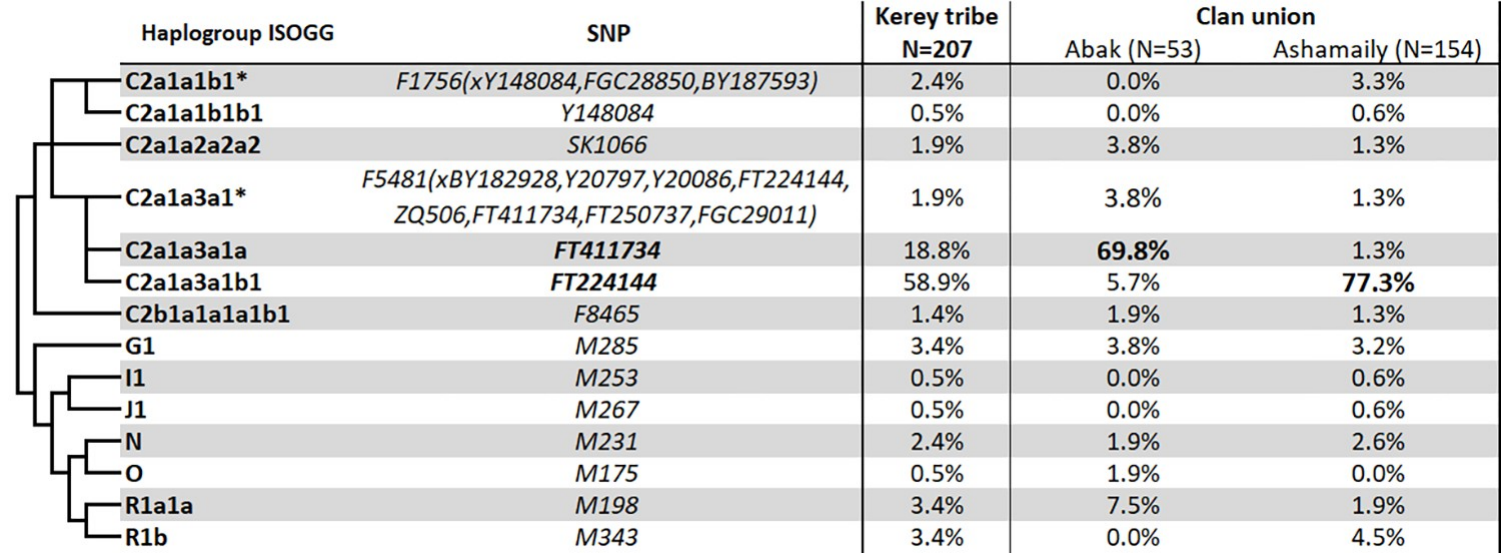
Frequencies of Y-chromosomal haplogroups in Kerey tribe and its clan unions.

Fig 2 and S1 Table display the haplogroup distributions of the Y-chromosome within the Kerey tribe of the Kazakh population. A total of eight core haplogroups were identified in our study, namely C2-M217, G1-M285, I1-M253, J1-M267, N-M231, O-M175, R1a-M198, and R1b-M343. The haplogroup C2-M217 is the most prevalent, accounting for 85.8% of the population. The subsequent haplogroups in the sequence are R1a (3.4%), G1 (3.4%), R1b (3.4%), and N (2.4%). The remaining portion constitutes less than one percent. The Kerey tribe’s genetic profile is mostly composed of various genetic components originating from ancient populations across Eurasia’s peripheral regions [26]. Additionally, there is a strong founder effect within the C2-M217 haplogroup.

The haplotype distributions of 23 Y chromosomal STRs in the Kerey tribe from the Kazakh population are presented in S1 Table. A total of 207 males were examined, resulting in the identification of 104 unique haplotypes, as detailed in S2 Table. There are a total of 83 unique haplotypes, with 21 of them being observed more than once. The haplotype with the highest frequency was seen in 46 instances. The haplotype in question is predominantly found in 96% of carriers who belong to the Taryshy lineage within the Ashamaily clan. The haplotype that occurs with the second highest frequency (18 occurrences) is likewise observed in 89% of the groups within the Ashamaily clan. The haplotype that occurs with the third highest frequency (17 occurrences) is exclusively found in groups belonging to the Abak clan. Additionally, we have identified a haplotype that is shared among five individuals as well as four haplotypes, each of which is shared among three individuals. Thirteen instances of haplotypes were observed on two separate occasions. In 81% of cases, similar haplotypes were found among the Ashamaily clan. The parameters haplotype diversity (HD = 0.068), discrimination capacity (DC = 50.2%), and haplotype match probability (HMP = 0.096) were determined. They point to the insufficient power of the PowerPlex Y23 System panel of loci used in differentiating close members of the clan along the male line for the Kerey tribe. S3 and S4 Tables present allele frequencies and forensic parameter values for 23 locus Y-STRs in the Kerey tribe. In single-copy loci, 113 alleles were found with a frequency of 0.005 to 0.942; in the DYS385a/b multilocus, 15 alleles were found with a frequency of 0.005 to 0.758. All loci are characterized by low levels of gene diversity (GD). Except for the DYS576 locus (GD 0.606), other rapidly mutating loci (DYS481, DYS533, DYS549, DYS570, DYS576, and DYS643) do not differ in diversity from standard single-copy loci. S5 Table presents the abnormal alleles found in the Kerey tribe. The observed genetic variations in the mentioned loci include deletion variants for DYS448, duplications at positions 16 and 17 for DYS19, and microvariants at position 18.2 for DYS458.

### Phylogenetics of haplogroup C2-M217 in the Kerey tribe

In prior research, it was shown that the C2-M217 haplogroup exhibits four primary branches among the Kazakh population. These branches are identified as C2a1a1b1-F1756, C2a1a2a2a-F5485, C2a1a3a-F4002, and C2b1a1a1a1a1a-M407. This study aimed to identify the specific sub-branches that are distinctive to the Kerey tribe and its clans, namely the Abak and Ashamaily.

The first branch, denoted as C2-F1756, had an exclusive presence solely among the Ashamaily clan, accounting for 3.9% of individuals. The field of citizen science, specifically in the context of C2-F1756, encompasses three distinct sub-branches denoted as Y148084, FGC28850, and BY187593. These sub-branches are characterized by the inclusion of individuals from Kazakhstan (https://discover.familytreedna.com/y-dna/C-F1756/tree). In our capacity as coordinators of the Kazakhstan Citizen Genealogical Science project, we obtained information regarding the gender of the volunteers who underwent Y-chromosome deep sequencing using the BigY method [41]. Therefore, it has been established that the sub branch C2-Y148084 is present within the Dulat clan, C2-FGC28850 is found in the Töre clan, and C2-BY187593 is identified in the Tabyn clan. The estimated time to the most recent common ancestor (TMRCA) for each sub-branch suggests that the individuals who initiated these branches existed during the 14th century AD. Upon doing a screening of the three branches in Ashamaily C2-F1756 carriers, it was determined that a single individual belongs to the C2-Y148084 sub-branch.

**Fig 3:**
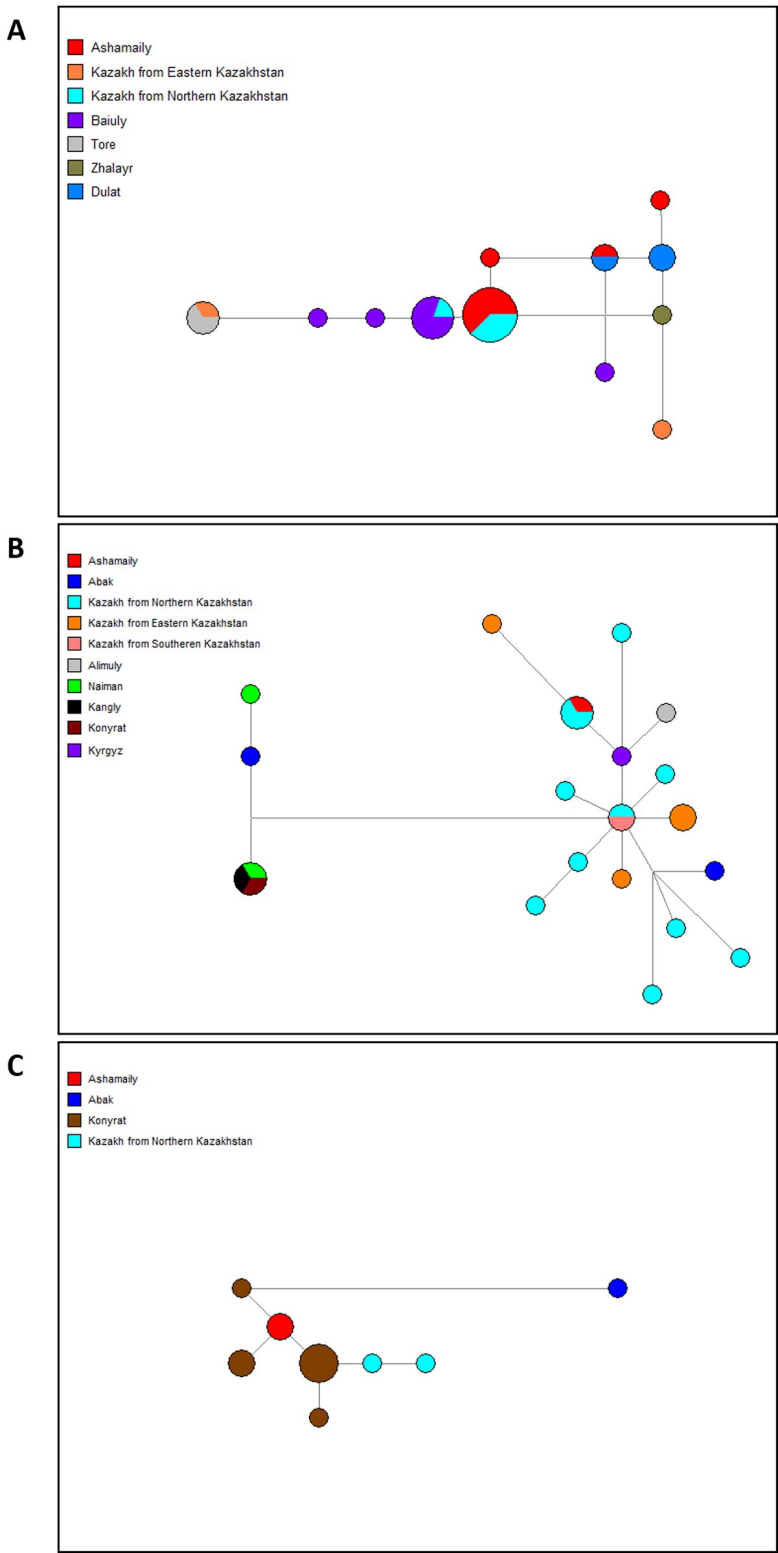
Median-joining network of Kazakh tribes based on 15 Y-STRs. A. Haplogroup С2-F1756; B. Haplogroup С2-SK1066; C. Haplogroup С2-F8465.

We conducted a search for haplotypes of C2-F1756 that were similar to those found in Ashamaily carriers. This search was performed within a comprehensive database of previously published haplotypes of Kazakhs that we had compiled. The database consisted of 3856 haplotypes, as determined by 17 Y-STR markers. One distinctive feature of the C2-F1756 haplogroup is the presence of a deletion at the DYS448 genetic locus. On the basis of the search results, a median network composed of 15 Y-STRs was constructed (Fig 3A). It emphasizes Ashamaily’s close connections with the Kazakh Yssyk clan of the Baiuly tribe, as well as the Zhalaiyr tribe, the Dulat clan of the Uissun tribe, and the Töre clan. On the median network, Kazakhs from Northern Kazakhstan (of an unknown clan affiliation to us) are associated with the Ashamaily, which correlates to the Kerey tribe’s establishment in this area of the country.

The second branch, denoted as C2-F5485, has been identified in the Abak clan at a frequency of 3.8% and in the Ashamaily clan at a frequency of 1.3%. In the preceding study, it was demonstrated that the C2-F5485 branch exhibits three distinct sub-branches (C2-SK1066, C2-Y15844, and C2-F12970), encompassing individuals of Kazakh descent from various lineages, as well as other Turkic and Mongolian speaking people. The C2-Y15552 lineage was identified as a founder effect for the Alimuly and Baiuly tribes within the C2-Y15844 lineage [39]. This analysis reveals that the identified lineage is absent in individuals that carry the C2-F5485 haplogroup in both the Abak and Ashamaily clans. The examination of three sub-branches has indicated that the Abak and Ashamaily under investigation serve as carriers of the C2-SK1066 sub-branch. This sub-branch in question had been previously observed within the Baiuly tribe, specifically within the Berish and Alasha clans. Through the utilization of citizen science, it has been ascertained that the C2-F9766 lineage, which falls within the C2-F12970 sub-branch, is also present within the Abak (Merkit lineage) and the Naiman tribe (https://discover.familytreedna.com/y-dna/C-F9766/tree). The chronological origin of their most recent common ancestor can be traced back to the 15th century.

The median network of 15 Y-STRs, as depicted in Fig 3B, illustrates the construction process using haplotypes obtained from carriers of Abak and Ashamaily C2-SK1066, as well as haplotypes that closely resemble them (within a maximum of three mutational steps). These haplotypes were sourced from a comprehensive database of Kazakhs, consisting of 3856 individuals and encompassing 17 Y-STRs. The majority of these haplotypes exhibit duplication at the DYS19 gene, resulting in the formation of two distinct clusters on the median network. Within a certain cluster denoted as α1, it is observed that an Abak belonging to the Sarbas lineage is categorized alongside the Naiman tribe. In the second category (α2), the Abak and the Ashamaily are categorized alongside the Kazakhs residing in eastern and northern regions of Kazakhstan. This classification aligns with the settlement patterns of the Kerey tribe in these specific areas. The median network does not exhibit any Ashamaily haplotype that possesses a deletion at the DYS448 locus. Among the Berish clan from the Baiuly tribe, only a single closely related haplotype was identified in his case.

The third branch, denoted as C2-M407, has been identified in the Abak clan at a frequency of 1.9% and in the Ashamaily clan at a frequency of 1.3%. The Kongyrat tribe has a significant prevalence of the C2-M407 branch, which accounts for 86% of the population [38]. Based on the information provided by the civil science data source (https://discover.familytreedna.com/y-dna/C-ZQ402/tree), it is observed that the Kongyrats are distinguished by their sub-branch C2-ZQ402, whereas the Ashamaily are associated with its fraternal sub-branch C2-F8465. The examination conducted on these sub-branches has indicated that the Abak and Ashamaily, which were the subjects of our study, are classified under the C2-F8465 sub-branch. We conducted a search for haplotypes of C2-F8465 that were similar to those found in carriers of Abak and Ashamaily within the comprehensive database of published haplotypes of Kazakhs that we compiled. The database included of 3856 individuals, as determined by 17 Y-STR markers. Based on the search results, a median network (Fig 3C) of 15 Y-STRs was made. The Ashamaily haplotypes form a cluster with some samples of Kongyrats and Kazakhs from northern Kazakhstan, and the haplotype of one Abak is distantly related to them.

### Genetic lineages of Abak and Ashamaily

C2-F4002, alternatively referred to as F3796, exhibits the highest prevalence (79.7%) within the Abak and Ashamaily clans. This particular lineage is associated with the renowned Star Cluster of Asia, which was initially identified through the use of Y-STR markers. It is believed that Genghis Khan and his male relatives on the paternal side serve as the progenitors of this lineage [42]. Nevertheless, a more in-depth analysis of the Y chromosome by sequencing techniques has revealed that the emergence of this particular lineage dates back to 550 BCE, which predates the era of Genghis Khan by a significant margin [43].

The subbranch C2-F5481 within the broader context of C2-F4002 garners significant interest. The presence of this genetic trait is observed in the majority of Central Asian populations. The Time to Most Recent Common Ancestor (TMRCA) has been estimated through extensive sequencing of the Y chromosome to be 860 CE. According to the data from the citizen science platform (source: https://discover.familytreedna.com/y-dna/C-F5481/tree), the C2-F5481 lineage currently has a minimum of three known distinct sub lineages (C2-Y12782, C2-BY182928, C2-F18202). The sub-lineage F18202 is the most diversified and comprises descendant lineages known as C2-ZQ31, C2-ZQ506, C2-FT411734, C2-MPB401, C2-FT250737, C2-FGC29011. With the exception of C2-ZQ31, identified within the Kyrgyz population, and C2-MPB401, present in the Afghan-Pakistani population, the remaining five lineages are associated with individuals from Kazakhstan. The sub-lineages C2-Y12782 and C2-BY182928 are also represented by samples from Kazakhstan. Moreover, C2-Y12782 further divides into three distinct branches: C2-Y20797, C2-Y20086, C2-FT224144. The genetic screening conducted on the Kerey tribe demonstrated that, among C2-F5481 lineages examined, lineage C2- FT224144 was found to be prevalent in the Ashamaily at a rate of 77.3%. Additionally, lineage C2-FT411734 was identified as a common lineage in the Abak clan, with a prevalence of 69.8%.

**Fig 4:**
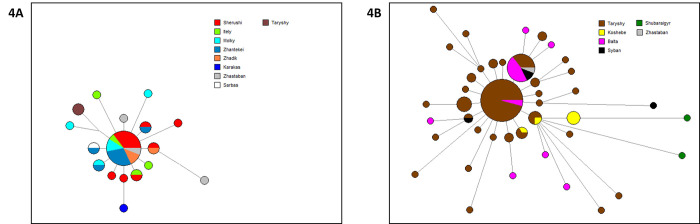
Median-joining network of Kerey tribe based on 21 Y-STRs. A. Haplogroup C2-FT411734; B. Haplogroup С2- FT224144.

The distinction between Abak and Ashamaily becomes more evident when examining their carriers within C2-FT411734, where the Ashamaily account for 5.1% and the Abak account for 94.9%. Similarly, within C2- FT224144, Abak carriers make up 2% while Ashamaily carriers make up 98%. The authors of the study [27] observed a distinction between the Abak and Ashamaily for the first time at the DYS448 locus, as indicated by the respective allelic values of 23 and 22. It was revealed that none of the carriers of the C2- FT224144 variant possess the DYS448 = 23 locus. Instead, 98.4% of carriers exhibit a DYS448 value of 22, while the remaining 1.6% exhibit a value of 21. In contrast, individuals carrying the C2-FT411734 haplotype frequently exhibit the DYS448 = 23 allele (89.7%). However, it is worth noting that a small proportion of haplotypes also possess the DYS448 = 22 (7.7%) and DYS448 = 24 (2.6%) alleles. It has been suggested that three individuals of Kazakh descent from China [27], who possess the DYS448 genetic marker with a value of 23, can be classified under the lineage F8949 (also known as C2-FT411734) within the broader lineage C2-F3796 (also known as C2-F4002). This particular lineage, F8949, has been observed to cluster together with the abacus lineage identified in the aforementioned study. The analysis of the C2-F5481 subclades demonstrated a distinct separation between the Abak and Ashamaily based on the Y-SNP markers C2-FT411734 and C2-FT224144, respectively. In our citizen science genealogical project, it was recently discovered that one individual from the Naiman tribe also belongs to the C-FT224144 lineage. Median networks were created for each lineage, depicting 21 short tandem repeats (STRs) (see Fig 4A and 4B). The haplotypes that were analyzed contained six loci that exhibited rapid mutation rates. However, these loci were not able to sufficiently differentiate and identify clusters peculiar to the species on median networks. Fig 4A displays that solely two samples belonging to the Taryshy Ashamaily population were classified within the Abak cluster. According to Fig 4B, the Ashamaily cluster is predominantly characterized by a significant abundance of the Taryshy lineage. Within the Abak, the Ashamaily cluster encompasses a solitary specimen from the Zhastaban lineage, as well as the exclusive pair of individuals from the minor Shubaraigyr lineage that are the subject of investigation in this paper. Hence, the identification of the alignment between clans and their respective relationships was unveiled, save for the Shubaraigyr clan and isolated instances.

According to Fig 4B, the Ashamaily cluster is predominantly characterized by a significant abundance of the Taryshy lineage. Within the Abak, the Ashamaily cluster encompasses a solitary specimen from the Zhastaban lineage, as well as the exclusive pair of individuals from the minor Shubaraigyr lineage that are the subject of investigation in this paper. Hence, the identification of the alignment between clans and their respective relationships was unveiled, save for the Shubaraigyr lineage and isolated instances.

The Time to the Most Recent Common Ancestor (TMRCA) based on deep Y-chromosome sequencing in the citizen science genealogical project for C2-FT224144 is approximately ~1139 CE (95% CI: 845–1369 CE), while for C2-FT411734 it is ~1337 CE (95% CI: 1072–1540 CE). TMRCA based on Y-STR markers coincides with the 12th-13th century CE. Thus, the TMRCA between the lineages does not support the notion that Abak and Ashamaily were brothers as presented in traditional Kazakh genealogy. They originate from a common ancestor, C-F5481, who lived around the mid-9th century.

### The Kerey’s paternal genetic heritage in the context of the Kazakh tribes

**Fig 5:**
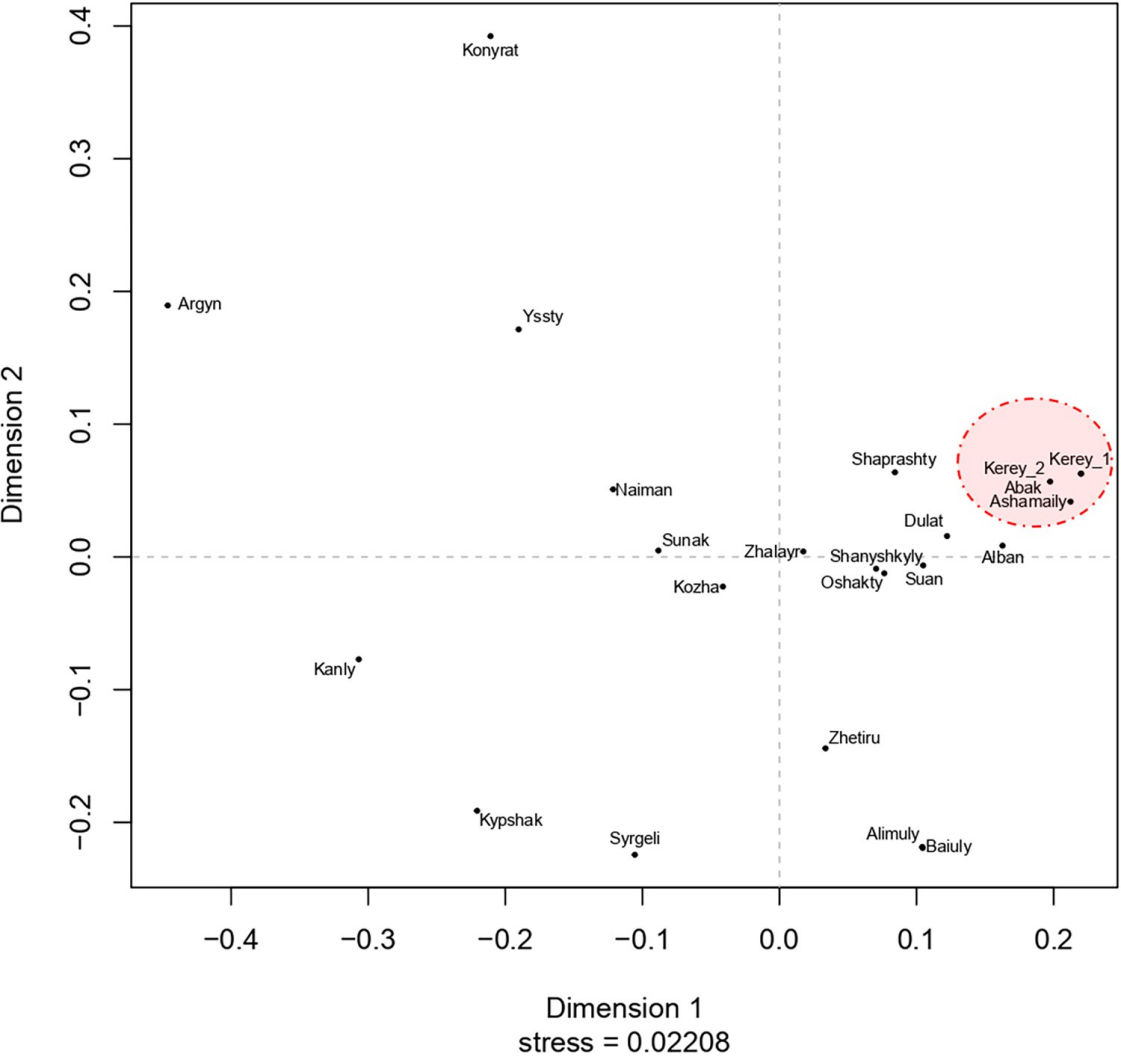
Multidimensional scale plot of Rst values estimated from 17 Y- STRs haplotypes among Kazakh tribes and clans.

Within the realm of historical and ethnographic literature, numerous hypotheses have been proposed concerning the interconnections between the Kerey tribe and other Kazakh tribes and clans, drawing upon the traditional genealogy of the Kazakh "shezhire". In order to ascertain the prevailing popularity among them, we conducted an analysis of the RST values between Kazakh tribal groups utilizing 17 short tandem repeat markers (as presented in S6 Table). Additionally, we generated a multidimensional scaling graph (Fig 5) to visually represent the obtained results.

The clan affiliations under investigation, namely the Abak and Ashamaily, are situated within the same cluster as the Kerey tribe samples examined in prior studies, specifically Kerey_1 [27] and Kerey_2 [28]. Simultaneously, no discernible distinctions were observed in the clan connections of the Abak and Kerey_2 originating from Aksu, located in the Xinjiang Uygur Autonomous Region. The reason for this phenomenon can be attributed to the widespread distribution of the Abak. The examined sample of the Abak clan association is found in the Bayan-Ulgii aimag of Mongolia (40%) and the Xinjiang Uygur Autonomous Region of China (36%).

In terms of genetic relatedness, the genera Alban (average RST—0.0592), Dulat (average RST: 0.0832), Suan (average RST—0.09852), and Shapyrashty (average RST—0.1297) exhibit the closest relationships to the generic counterparts Abak and Ashamaily. The Argyn, Kongyrat, and Kangly tribes are considered to be the most geographically remote, as indicated by their respective average RST values of 0.6351, 0.6189, and 0.5972.

The Uissun tribe encompasses the Alban, Dulat, Suan, and Shapryshaty clans. Based on the ethnographic data [44], the initial prevailing hypothesis posits a relationship between the Kerey tribe and the Uissun tribe. Simultaneously, according to one account, it was the progenitor Abak who sprang from the Uissun tribe [18]. It was observed that the C2-Y12782 lineage had a rather high frequency of occurrence throughout the Uissun tribe, accounting for around 31% of the population [38]. Therefore, the phylogenetic data provide evidence supporting the connection between the Kerey and Uissun tribes. However, they contradict the hypothesis that the Abak originate from the Uissun tribe, as the presence of the C2-Y12782 lineage, specifically the sub-lineage C2-FT224144, is indicative of the Ashamaily clan affiliation.

Based on the second prominent hypothesis, which draws from a comparative analysis of the tribe’s tamgas [44], it is suggested that there exists a relationship between the Kerey and Sirgeli tribes. Nevertheless, it is worth noting that the genetic distances between the Abak and Ashmaily clans and the Sirgeli tribe exhibit a considerable degree of divergence, as indicated by the average RST value of 0.4825. This pattern is further supported by the visualization of the multidimensional scaling (MDS) plot. The Sirgeli tribe exhibits a high prevalence (71%) of the N-M231 Y-chromosome haplogroup [9,38]. In contrast, the Kerey tribe demonstrates a significantly lower occurrence (2.4%) of this haplogroup. Haplogroup N-M231 exhibits a notable prevalence within the Uak tribe, accounting for around 46% of their genetic composition. According to a prominent hypothesis, this haplogroup demonstrates a strong ancestral connection with the Kerey tribe. This conjecture is supported by oral folklore, where the Kerey-Uak collective is referred to as "born together" [15]. Based on ethnographic data, the Sarcha, Sarman, and Zhansary clans are identified as originating from the Kerey tribe [44]. Regrettably, the available material pertaining to the clan level of the Uak tribe is insufficient to substantiate the aforementioned accounts. The sample size of the Uak tribe, consisting of only 11 people, was deemed insufficient for inclusion in the calculation of the RST and hence, it is not shown on the Multidimensional Scaling (MDS) graph. Nevertheless, the prevalence of haplogroup C2a1a3a-F4002 within the Uak tribe, at 18%, is not sufficiently substantial. This finding tends to negate the notion of a tight affiliation between the Kerey and Uak tribes, rather than providing support for the hypothesis derived from folklore.

One of the prevailing hypotheses, which has gained popularity, posits a single origin for the Kerey tribe and the Kereyt clan. This argument is supported by the consonance observed in their names [15]. Currently, there is limited knowledge regarding Y-chromosome variants within the Kereyit clan. Specifically, four individuals have been identified with such variants, [9,35,39]. These variants include E-M35, N-M231, and C2-M48. The haplotypes observed for the 17 Y-STRs do not conform to the expected patterns found within the Kerey tribe. Nevertheless, further investigation into the Kereyit population is necessary in order to definitively confirm this concept and gain a comprehensive understanding of its association with the medieval Kereyit tribe. Presently, the Kereyit clan is encompassed inside the Zhetiru tribe, alongside six additional clans. Notably, the Tabyn and Tama clans are prominently featured in the available samples. The average RST value of the Kerey tribe is 0.2619, which indicates a significant genetic distance from the Zhetiru tribe. The MDS chart further supports this genetic divergence. The Zhetiru tribe is situated in close proximity to the Alimuly (RST—0.0851) and Baiuly (RST—0.0885) tribes.

The predominant Y-chromosome haplogroup observed among the Alimuly and Baiuly tribes is C2-M48, with frequencies of 77% and 71%, respectively [39]. Ethnographic data suggests that the Adai clan of the Bayuly tribe and the Törtkara clan of the Alimuly tribe can be traced back to the Kerey tribe [15]. The determination of haplogroup frequencies at the clan level demonstrates a notable prevalence of the C2-M48 haplogroup (85%) among the Adai clan, whereas the Törtkara clan exhibits a relatively lower frequency of C2-M48 (39%) and Q-M242 (32%). Within the Kerey tribe, the presence of C2-M48 is infrequent, accounting for a mere 1.9% of the population. Conversely, the Q-M242 lineage is entirely absent among the tribe’s members.

An alternative hypothesis is that the Karakerey clan of the Naiman tribe may have ancestral ties to the Kerey tribe, based on the consonance observed between their respective names [11]. The Naiman tribe has a prevalent haplogroup on the Y chromosome, specifically O-M175, which is found in 43% of individuals. This haplogroup is also observed in the Karakerey clan, with a frequency of 70% [9]. Within the Kerey tribe, the occurrence of this particular phenomenon is exceedingly infrequent, with a prevalence rate of barely 0.5%. The multidimensional scaling (MDS) graph shows that the genetic differences between the Abak and Ashamaily clans and the Naiman tribe are quite different (RST = 0.3632).

Ultimately, the analysis of this data facilitated the determination of the Kerey tribe’s position within the genetic landscape of Kazakh tribes. Furthermore, it served to dismiss several prevalent historical and anthropological hypotheses regarding their origins, precluding their further consideration in the discourse surrounding the formation of the Kazakh population. The identified clan-specific markers, namely C2-FT411734 and C2- FT224144, which are associated with the generic associations Abak and Ashamaily, respectively, can be utilized in forensic medical examinations. These markers are particularly useful in determining the biogeographical origin of individuals in the male lineage. Additionally, they can aid in the assessment of rapid-mutation Y-STRs, necessitating the expansion of the panel for generic groups in Central Asia.

## Conclusions

The tribe Kerey has been found to exhibit two prevalent phylogenetic lineages of the Y-chromosome: the Abak clan is characterized by the C2-FT411734 lineage, while the Ashamaily clan is associated with the C2- FT224144 lineage. The estimation of the time to the most recent common ancestors for the Abak and Ashamaily clans indicate that they do not share a sibling relationship, contrary to what is stated in traditional genealogy. Rapid-mutation Y-STRs (DYS576, DYS481, DYS549, DYS533, DYS570, DYS643) did not exhibit sufficient power of differentiation among lineages within the Abak and Ashamaily clans. Prevailing historical and ethnographic hypotheses regarding the kinship of the Kerey tribe with other clans have been genetically verified. The construction of the genealogy of the Kerey tribe to the stepfather of Genghis Khan has been called into question.

## Supporting information

S1 Checklist Inclusivity in global research. (DOCX)

S1 Table The haplotype distributions of 23 Y-chromosomal STRs in the Kerey tribe from the Kazakh population (N = 207). (XLSX)

S2 Table The haplotype frequencies of 23 Y-chromosomal STRs in the Kerey tribe from the Kazakh population (N = 207). (XLSX)

S3 Table Allele frequencies and Forensic parameters values for 21 single-locus Y-STRs in the Kerey tribe from the Kazakh population (N = 207). (XLSX)

S4 Table Allelic combination frequencies and Forensic parameters values for DYS385a/b in the Kerey tribe from the Kazakh population (N = 207). (XLSX)

S5 Table Allelic micro-variants detected in the Kerey tribe from the Kazakh population. (XLSX)

S6 Table Pairwise genetic distance (RST) between Geographical Kazakh populations and Kazakh clans on 17 Y-STRs. (XLSX)
